# The *P*. *falciparum* CSP repeat region contains three distinct epitopes required for protection by antibodies *in vivo*

**DOI:** 10.1371/journal.ppat.1010042

**Published:** 2021-11-08

**Authors:** Yevel Flores-Garcia, Lawrence T. Wang, Minah Park, Beejan Asady, Azza H. Idris, Neville K. Kisalu, Christian Muñoz, Lais S. Pereira, Joseph R. Francica, Robert A. Seder, Fidel Zavala

**Affiliations:** 1 Johns Hopkins Bloomberg School of Public Health, Department of Molecular Microbiology and Immunology, Malaria Research Institute, Baltimore, Maryland, United States of America; 2 Vaccine Research Center, National Institute of Allergy and Infectious Diseases, National Institutes of Health, Bethesda, Maryland, United States of America; 3 Medical Technology Department, Faculty of Health Science, University of Antofagasta, Antofagasta, Chile; Francis Crick Institute, UNITED KINGDOM

## Abstract

Rare and potent monoclonal antibodies (mAbs) against the *Plasmodium falciparum* (Pf) circumsporozoite protein (CSP) on infective sporozoites (SPZ) preferentially bind the PfCSP junctional tetrapeptide NPDP or NVDP minor repeats while cross-reacting with NANP central repeats *in vitro*. The extent to which each of these epitopes is required for protection *in vivo* is unknown. Here, we assessed whether junction-, minor repeat- and central repeat-preferring human mAbs (CIS43, L9 and 317 respectively) bound and protected against *in vivo* challenge with transgenic *P*. *berghei* (Pb) SPZ expressing either PfCSP with the junction and minor repeats knocked out (KO), or PbCSP with the junction and minor repeats knocked in (KI). *In vivo* protection studies showed that the junction and minor repeats are necessary and sufficient for CIS43 and L9 to neutralize KO and KI SPZ, respectively. In contrast, 317 required major repeats for *in vivo* protection. These data establish that human mAbs can prevent malaria infection by targeting three different protective epitopes (NPDP, NVDP, NANP) in the PfCSP repeat region. This report will inform vaccine development and the use of mAbs to passively prevent malaria.

## Author summary

Human monoclonal antibodies are a promising approach for preventing malaria. Highly potent human antibodies show preferential binding to the junction or minor repeat regions of the circumsporozoite protein (CSP) of *P*. *falciparum* and cross-react to repetitive (NANP) repeats. The requirement for these binding sites for mediating protection *in vivo* remains unknown. Here, using transgenic *P*. *berghei* parasites expressing PfCSP containing deletions of these junctional or minor epitopes, or PbCSP containing additions of these PfCSP epitopes, we demonstrate these epitopes are necessary and sufficient to mediate protection and don’t require cross-reactivity to the NANP repeats. Our findings establish a mechanism of antibody mediated protection *in vivo* to prevent malaria and provide the rationale for multi-epitope vaccines to increase the breadth of the antibody responses.

## Introduction

Malaria is a leading cause of death in infants and small children around the world. Infection caused by *Plasmodium falciparum* (Pf) in Africa is primarily responsible for the mortality. Public health measures and drug treatments have tremendously reduced morbidity and mortality, but these efforts have plateaued. Thus, additional interventions are urgently needed to control malaria [1].

Developing a malaria vaccine that provides durable protection against clinical disease and completely prevents infection will be critical for controlling and eliminating malaria. RTS,S, which targets the Pf circumsporozoite protein (CSP) expressed on the surface of sporozoites (SPZ), is a leading malaria vaccine candidate undergoing Phase III clinical trials in malaria-endemic areas. RTS,S is a virus-like particle consisting of a portion of PfCSP genetically fused to Hepatitis B surface antigen and is administered with the adjuvant AS01 [2]. Results from several Phase III trials indicate that three immunizations with RTS,S/AS01 induce anti-PfCSP antibodies that confer ~50% protection against clinical disease after 1 year and ~25% after 3 years [3]. While these results are encouraging and show that antibodies against PfCSP can be protective, a more efficacious vaccine is needed to bolster efforts to control malaria.

A second approach for preventing malaria is passive transfer of highly potent human monoclonal antibodies (mAbs) against PfCSP. This intervention could allow for high-level malaria prevention for up to 6 months with a single administration of a PfCSP-specific mAb in humans [4]. For both active and passive immunization, it will be important to identify the precise PfCSP epitopes that mediate protection after binding of mAbs *in vivo*.

Several studies had shown that most inhibitory anti-PfCSP polyclonal antibodies and mAbs recognize (NANP)_n_ major repeats in the central repeat (CR) domain of PfCSP [5,6]. Such is the case for 317, a potent mAb isolated from a volunteer immunized with RTS,S that recognizes NPNA major repeat motifs in the CR domain [6]. However, recent studies using human mAbs isolated from individuals immunized with irradiated PfSPZ indicate that some neutralizing mAbs–while cross-reacting with (NANP)_n_−preferentially recognize related but different repeat epitopes. For instance, mAb CIS43 preferentially binds the junction region (JR) ADGNPDPNANPNVDP located at the junction of the N-terminus and CR domain [7,8]. Another highly protective mAb, L9, preferentially recognizes the NPNV minor repeat motifs associated with minor repeats (MR) NANPNVDPNANPNVDP located immediately after JR [9].

A shared feature of these three highly potent mAbs is that they bind recombinant PfCSP in two distinct events (termed “two-step binding”) by isothermal titration calorimetry [7,9]. For CIS43 and L9, the first binding event shows high-affinity binding to their respectively preferred JR or MR epitopes whereas the second binding event shows lower-affinity, multivalent binding to CR epitopes. While CIS43, L9, and 317 preferentially bind JR, MR and CR epitopes, respectively, they do promiscuously bind all three epitopes in the repeat region (S1 Table). This extensive cross-reactivity has raised questions regarding the extent to which the *in vivo* protection mediated by these mAbs is strictly due to recognition of their preferred repeat epitopes or if it is influenced by binding to their non-preferred repeat epitopes [10,11].

To determine whether recognition of their preferred JR, MR, or CR epitopes is necessary and sufficient for these three highly potent human mAbs to neutralize SPZ *in vivo*, we generated a set of transgenic *P*. *berghei* (Pb) parasite lines expressing modified CSP. Some of these parasite lines express PfCSP lacking the JR, MR or JR+MR (knock-out, KO parasites) while other lines express a modified PbCSP containing the relevant (JR and JR+MR) PfCSP epitopes (knock-in, KI parasites). The *in vitro* binding and *in vivo* protection of CIS43, L9, and 317 against these transgenic SPZ lines were assessed. Overall, the data presented here validate the JR, MR, and CR epitopes as distinct antigenic determinants in the PfCSP repeat region respectively required by CIS43, L9, and 317 to potently mediate protection *in vivo*. These data will inform the design of future PfCSP-based vaccines and guide clinical development of potent mAbs for passive malaria prevention.

## Results

### Generation of transgenic parasite lines

**Fig 1:**
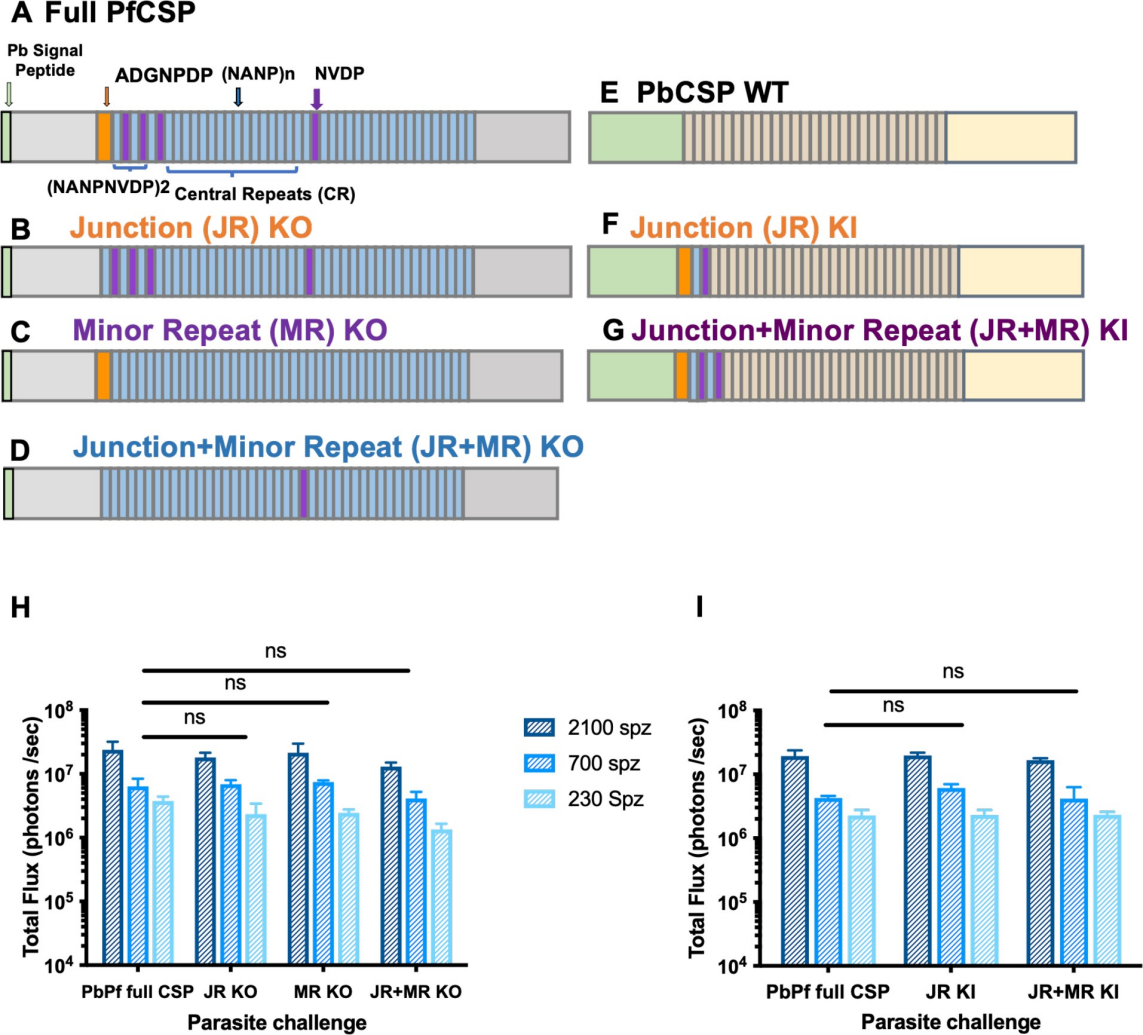
Schematics of CSP versions expressed by Pb transgenic parasite lines and their infectivity in mice. (A) Pb expressing full-length PfCSP (PbPf full CSP). The Pb signal peptide (green), PfCSP N- and C-termini (grey), junction region ADGNPDP (JR, orange), 4 NVDP minor repeats (MR, purple), and 38 central major repeats (CR, blue) are indicated. (B) Pb expressing PfCSP with ADGNPDP deleted (JR KO). (C) Pb expressing PfCSP where the 4 NVDP were changed to NANP (MR KO). (D) Pb expressing PfCSP with ADGNPDP deleted and 3 of the 4 NVDP changed to NANP (JR+MR KO). (E) Pb expressing wild-type PbCSP. The N-terminus (green), repeat region (grey), and C-terminus (yellow) are depicted. (F) Pb expressing PbCSP containing the PfCSP JR sequence ADGNPDPNANPNVDP (JR KI). (G) Pb expressing PbCSP containing the PfCSP JR and MR sequences ADGNPDPNANPNVDPNANPNVDP (JR+MR KI). (H-I) Infectivity of the different transgenic parasite lines depicted in A-G, evaluated by challenging C57BL/6 mice with indicated amounts of SPZ and measuring parasite bioluminescence (total flux, photons/sec) in the liver 42 hours later. No statistically significant differences were found in the infectivity of the different parasite lines (Kruskal Wallis).

To assess the distinct PfCSP epitope(s) required by human mAbs to mediate protection against SPZ challenge *in vivo*, genetically engineered Pb transgenic parasite lines with either KO deletions of epitopes in the PfCSP gene or KI additions of PfCSP epitopes to the endogenous PbCSP gene were created. As a positive control, we utilized a published transgenic Pb line where the entire endogenous PbCSP was replaced with full-length PfCSP (PbPf full CSP, Fig 1A). Importantly, these transgenic PbPf full CSP SPZ had identical infectivity to wild-type PbSPZ [12].

To generate the transgenic Pb KO lines, the endogenous PbCSP was replaced with modified PfCSP versions lacking the JR (Junction KO, Fig 1B), MR (Minor Repeat KO, Fig 1C), or JR+MR (Junction + Minor Repeat KO, Fig 1D) using previously described procedures [13]. For transgenic Pb KI lines, the endogenous PbCSP (PbCSP WT, Fig 1E) was replaced with modified versions of PbCSP containing short inserts corresponding to the JR (Junction KI, Fig 1F) and JR+MR (Junction + Minor Repeat KI, Fig 1G). The modified CSP genes expressed by all transgenic Pb lines were confirmed by PCR amplification followed by sequencing (S2 Table).

We first established whether the KO/KI transgenic parasite lines had altered *in vivo* infectivity compared to the positive control PbPf full CSP SPZ. Importantly, all parasite lines generated in this study express luciferase, which enables the monitoring of their infective capacity in mice by measuring bioluminescence [12]. Different numbers of SPZ from the KO/KI lines were injected intravenously (i.v.) and parasite liver burden was evaluated 42 hours later (Fig 1H and 1I). All the newly generated transgenic KO/KI SPZ lines displayed infectivity comparable to PbPf full CSP SPZ. In addition, all the parasite lines demonstrated similar infections in mosquitoes and generated comparable numbers of oocysts and SPZ in salivary glands (S3 Table). These data show that removal of the JR and MR regions from the PfCSP transgene in Pb, or addition of such sequences to the endogenous PbCSP, does not affect the infectivity of transgenic *P*. *berghei* parasites in mice or development in mosquitoes.

### Binding of PfCSP mAbs to transgenic SPZ lines *in vitro*

**Fig 2:**
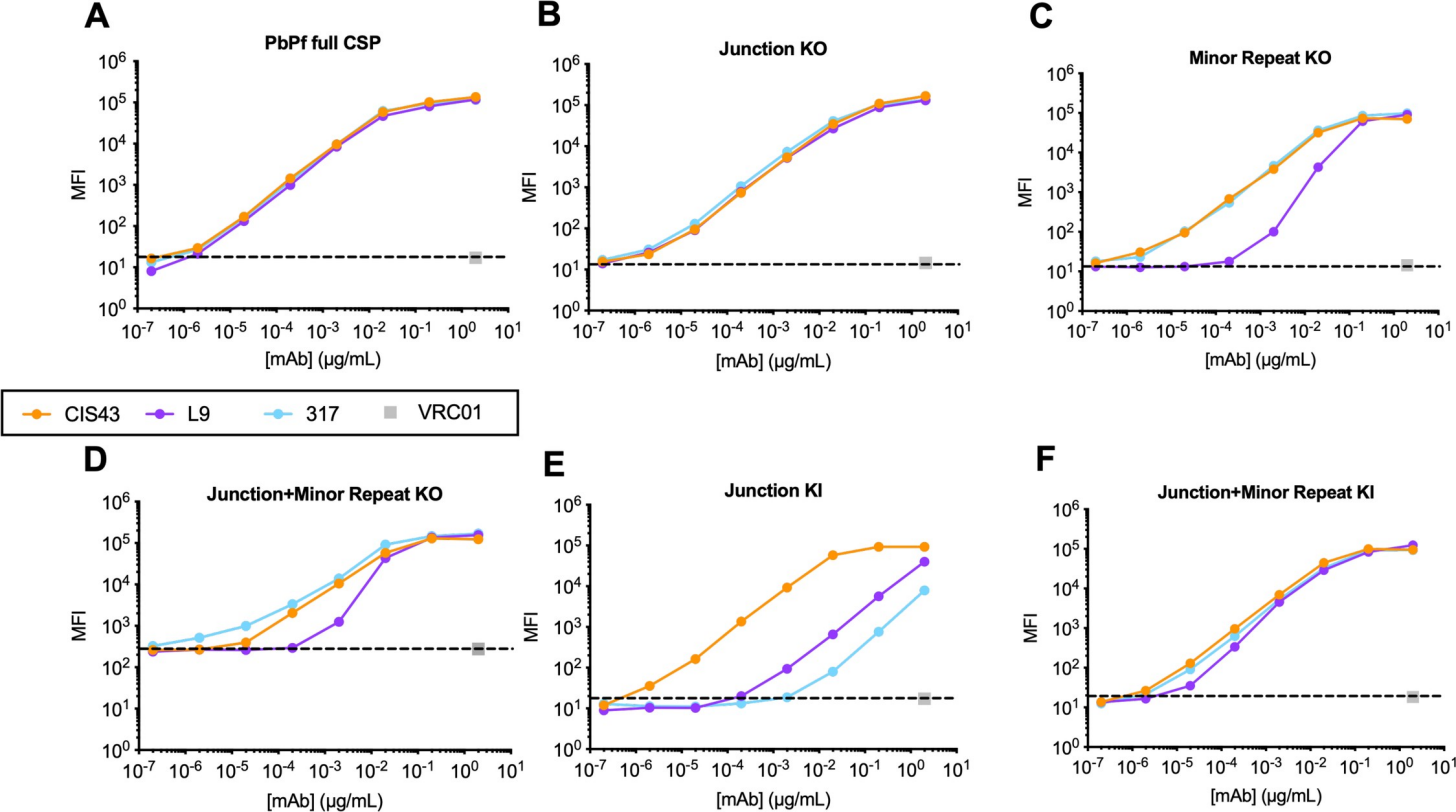
Flow cytometry analysis of PfCSP mAbs binding to transgenic SPZ lines. Flow cytometric measurement of various concentrations (2.0–2 x 10^−7^ μg/mL) of mAbs CIS43, L9, and 317 binding to freshly isolated SPZ expressing (A) PbPf full CSP, (B) Junction KO, (C) Minor Repeat KO, (D) Junction and Minor Repeat KO, (E) Junction KI, and (F) Junction and Minor Repeat KI. The anti-HIV-1 mAb, VRC01 was included as an isotype control. mAb binding is expressed as median fluorescence intensity (MFI).

To define the binding pattern of PfCSP mAbs to the transgenic KO/KI SPZ lines, flow cytometry was used to measure the binding of anti-JR, anti-MR, and anti-CR mAbs (CIS43, L9 and 317 respectively; S1 Table) to each SPZ line. As a positive control, all mAbs tested had high and comparable binding to PbPf full CSP SPZ (Fig 2A). CIS43, L9, and 317 all bound the JR KO SPZ similarly to PbPf full CSP SPZ (Fig 2B). This was an unexpected finding for CIS43, as a previous report had shown that the binding affinity of CIS43 for recombinant PfCSP lacking JR was substantially less than for wild-type PfCSP [7]. This discrepancy might be attributable to CIS43 binding to MR and the multiple NANP motifs associated with CR epitopes, as indicated in S1 Table [9].

L9 displayed reduced, though detectable, binding to MR KO SPZ; conversely, CIS43 and 317 bound the MR KO SPZ equivalently to PbPf full CSP (Fig 2C). These data are consistent for all three mAbs and confirm the preference of L9 for MR epitopes and its cross-reactivity with CR epitopes on recombinant PfCSP [9]. Similarly, L9 binding was reduced against JR+MR KO SPZ (Fig 2D), though to a lesser extent than MR KO SPZ, likely due to the single NVDP remaining on PfCSP in the JR+MR KO SPZ (Fig 1D).

To complete the analysis, we measured the binding of these three mAbs to KI SPZ expressing JR and MR inserted into the endogenous PbCSP. Consistent with the identification of CIS43 as an anti-JR mAb [7], CIS43 strongly bound JR KI SPZ comparably to PbPf full CSP SPZ while L9 and 317 showed lower, but detectable, binding (Fig 2E). Interestingly, all three mAbs demonstrated high binding to JR+MR KI SPZ that was comparable to PbPf full CSP SPZ (Fig 2F). The 50% binding of the mAbs to each parasite was calculated and the results are shown in S4 Table. Together, these results highlight these three mAbs’ complex pattern of preferential and cross-reactive binding to the JR, MR, and CR epitopes in the PfCSP repeat region.

### Effect of PfCSP mAbs on *in vitro* motility of transgenic SPZ lines

We first evaluated the ability of CIS43, L9, and 317 to immobilize the different transgenic SPZ lines *in vitro* as an initial test of these mAbs’ potential dependence on the JR, MR, and CR epitopes to neutralize SPZ. Motility is a critical function for SPZ pathogenesis, as it enables their migration through the skin to invade blood vessels and their egress from the bloodstream to invade hepatocytes in the liver [14,15]. Anti-CSP antibodies have been shown to immobilize SPZ and thereby inhibit their *in vivo* infectivity [16,17].

**Fig 3:**
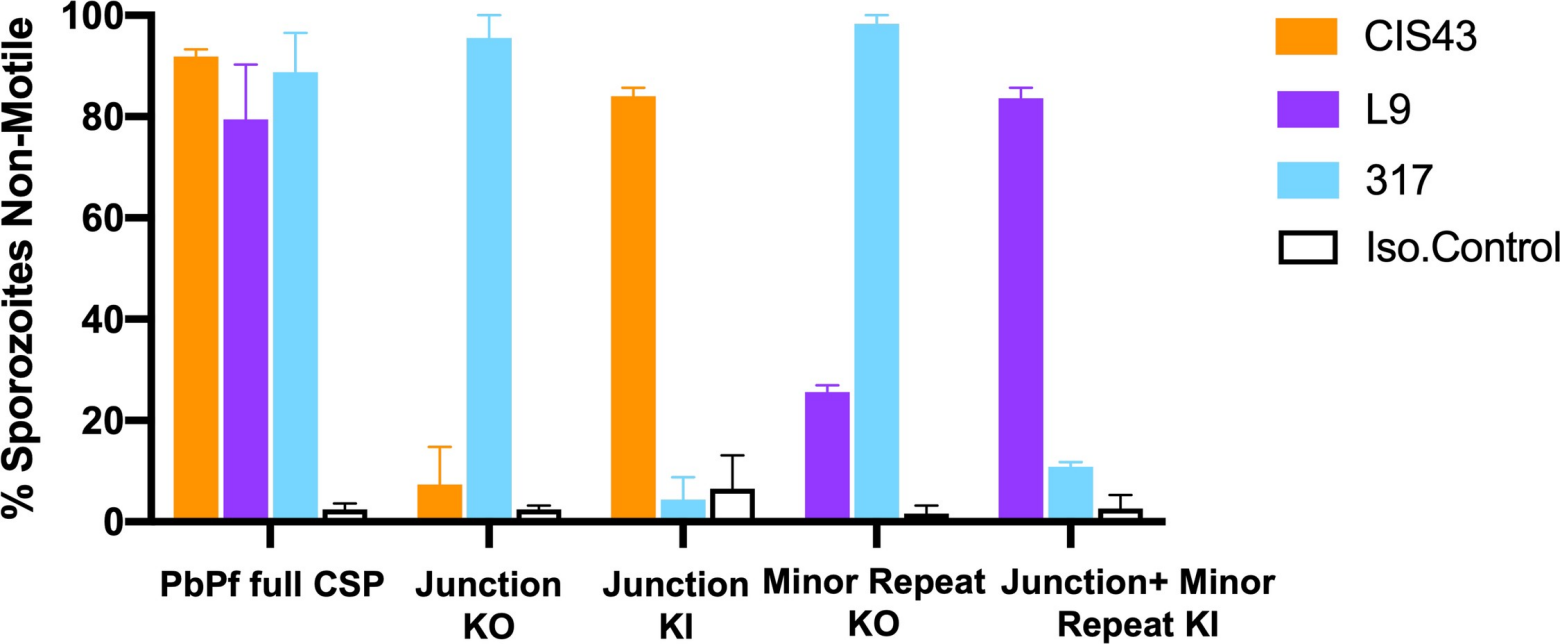
Quantitative analysis of PfCSP mAbs immobilizing SPZ *in vitro*. Effect of mAbs on *in vitro* motility of freshly isolated transgenic KO/KI SPZ lines. The percentage of SPZ that became non-motile after incubation with CIS43 (50 μg/ml) or L9, 317 or isotype control (25 μg/ml each) for 15 minutes at 37°C was determined using time-lapse microscopy. The bar graphs show the mean and standard error of the percentage of non-motile SPZ from two independent experiments.

As a positive control, CIS43, L9, and 317 showed high (80–90%) immobilization of PbPf full CSP SPZ after 15 minutes of *in vitro* incubation (Fig 3). However, CIS43 and L9 respectively showed limited immobilization of JR KO and MR KO SPZ while 317 fully immobilized both SPZ lines (Fig 3), which contain its preferred CR epitopes. In contrast, CIS43 and L9 respectively immobilized JR KI and JR+MR KI SPZ while 317 had no effect on either SPZ line, which lack its preferred CR epitopes (Fig 3). Together, these data show that the JR, MR, and CR epitopes are necessary and sufficient for CIS43, L9, and 317 to respectively immobilize SPZ *in vitro*. Importantly also, these results indicate that antibody binding to a single epitope is sufficient to immobilize sporozoites.

### *In vivo* protection by PfCSP mAbs against i.v. challenge with transgenic SPZ lines

**Fig 4:**
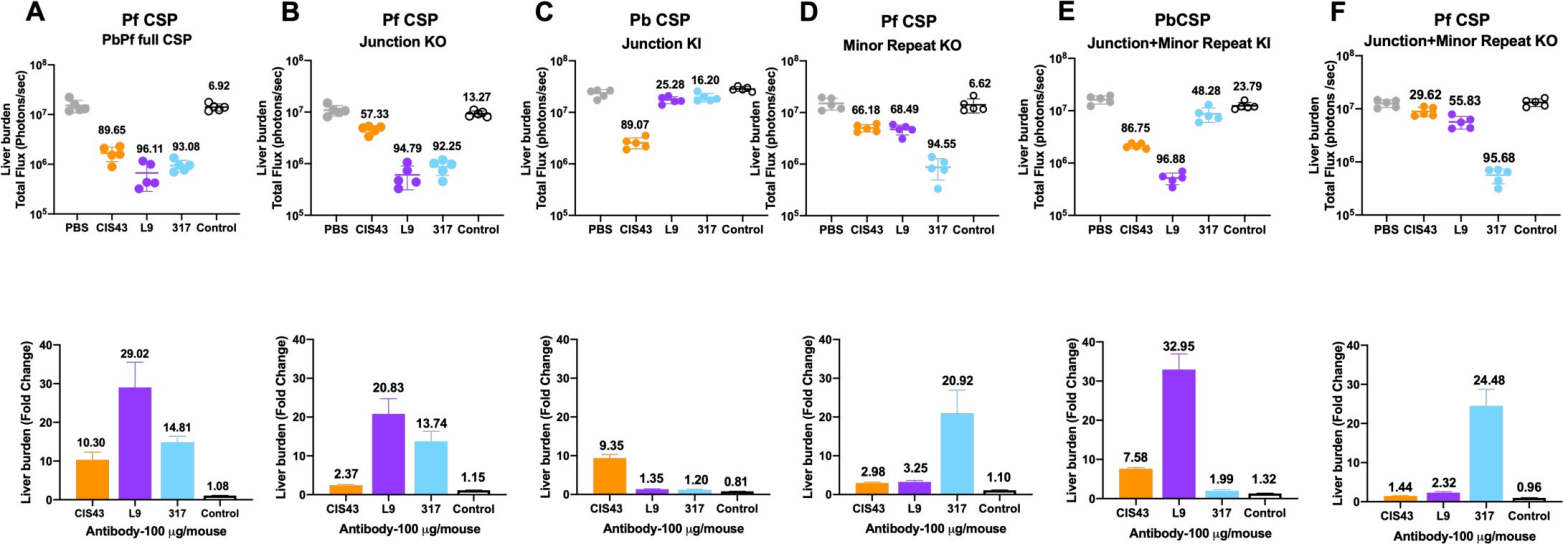
PfCSP mAb-mediated liver burden reduction in mice challenged i.v. with transgenic SPZ lines. C57BL/6 mice (5/group) were injected i.v. with PBS or 100 μg of mAbs CIS43, L9, 317, or isotype control and i.v. challenged 16 hours later with 2,000 transgenic SPZ expressing A) PbPf full CSP, B) Junction KO, C) Junction KI, D) Minor Repeat KO, E) Junction+ Minor Repeat KI, and F) Junction + Minor repeat KO. Upper panels: parasite bioluminescence in the liver (total flux, photons/sec); lines indicate geometric mean and geometric standard deviation. The percent reduction, calculated in relation to the PBS control mice, is noted above each mAb group. Lower panels: fold reduction in liver burden mediated by each mAb, calculated as the ratio of the total flux in the PBS control mice divided by total flux in each mAb group.

Given the complex binding pattern of mAbs CIS43, L9, and 317 to the transgenic KO/KI SPZ lines *in vitro* (Fig 2), we next assessed whether mAb-mediated reduction in parasite liver burden following i.v. challenge with the KO/KI SPZ was dependent on binding to their preferred JR, MR, or CR epitopes or cross-reactivity with other repeat epitopes. As a positive control, mice that received 100 μg of CIS43, L9 or 317 mAbs i.v. 16 hours before challenge with PbPf full CSP SPZ showed 10-29-fold liver burden reductions (90–96% inhibition) (Fig 4A), consistent with prior studies [6,7,9]. As a negative control, none of these mAbs protected against challenge with wild-type PbSPZ, which do not contain any PfCSP sequences (S1 Fig).

Next, we assessed the abilities of CIS43, L9, and 317 to protect against i.v. challenge with the JR KO/KI SPZ lines. While L9 and 317 protection against the JR KO SPZ was comparable to PbPf full CSP SPZ, CIS43 only reduced JR KO SPZ liver burden by 2.3-fold (57% inhibition) (Fig 4B), a significantly diminished inhibitory effect compared to PbPf full CSP SPZ (10-fold reduction, Figs 4A and S2A). In contrast, CIS43-mediated protection was largely restored (9.3-fold reduction) against JR KI SPZ while L9 and 317 had very low inhibition against these SPZ, which lack their preferred MR and CR epitopes (Figs 4C and S2B). Together, these results indicate that the JR epitope is necessary and sufficient for CIS43 to fully protect against i.v. SPZ challenge.

A similar *in vivo* protection analysis of CIS43, L9, and 317 was performed with MR KO SPZ. L9-mediated protection was substantially reduced against the MR KO (3.2-fold reduction, Fig 4D) compared to PbPf full CSP SPZ (29-fold reduction Figs 4A and S2C). Notably, the inhibitory activity of CIS43 was also reduced against MR KO SPZ (3-fold, 66% inhibition; Figs 4D and S2C), likely due to the removal of DPNA motifs associated with MR epitopes (S2 Table) that are recognized by CIS43 [7,9]. As expected, the inhibitory effect of 317 was similar against the MR KO and PbPf full CSP SPZ. These results show that L9 and CIS43 respectively require NPNV and DPNA motifs associated with MR epitopes (NA|NPNV|DPNA|NPNV|DP) to effectively neutralize SPZ *in vivo*.

Having shown that the JR and MR epitopes are critical for *in vivo* protection mediated by CIS43 and L9, we next determined the inhibitory capacity of these mAbs against JR+MR KI SPZ. CIS43 and L9 mediated high levels of protection (7.5- and 32.9-fold reduction respectively) against the JR+MR KI SPZ that were comparable to protection against PbPf full CSP SPZ (Figs 4E and S2D). Conversely, 317 exhibited modest inhibition (1.9-fold reduction) against these SPZ. Together, these results show that the MR epitope is necessary and sufficient for L9 to potently neutralize SPZ *in vivo*.

Finally, the protective efficacy of these mAbs against JR+MR KO SPZ was evaluated. CIS43 and L9 had modest inhibitory effects (1.4- and 2.3-fold reduction) on the JR+MR KO SPZ (Figs 4F and S2E). In contrast, 317 reduced liver burden by 24-fold (96% inhibition) in mice challenged with JR+MR KO SPZ (Fig 4F), which was comparable to protection observed in mice challenged with PbPf full CSP SPZ (Figs 4A and S3E). When considered alongside the JR and JR+MR KI data indicating that 317 has no inhibitory effect on parasites lacking CR (Fig 4C and 4E), these data confirm that 317 requires the CR epitope to inhibit SPZ *in vivo*.

As all preceding studies were performed using 100 μg/mouse of CIS43, L9, and 317, we also performed dose response experiments using 300 and 30 μg/mouse of each mAb (S3 Fig). These titration experiments confirmed the overall pattern of protection conferred by CIS43, L9, and 317 against the transgenic KO/KI SPZ lines. Collectively, these data provide *in vivo* evidence that CIS43, L9, and 317 require their preferred JR, MR, and CR epitopes to potently protect against i.v. SPZ challenge and that their extensive cross-reactivity with secondary repeat epitopes contributes minimally to *in vivo* protection.

### Protection by PfCSP mAbs against mosquito bite challenge with transgenic SPZ lines

**Fig 5:**
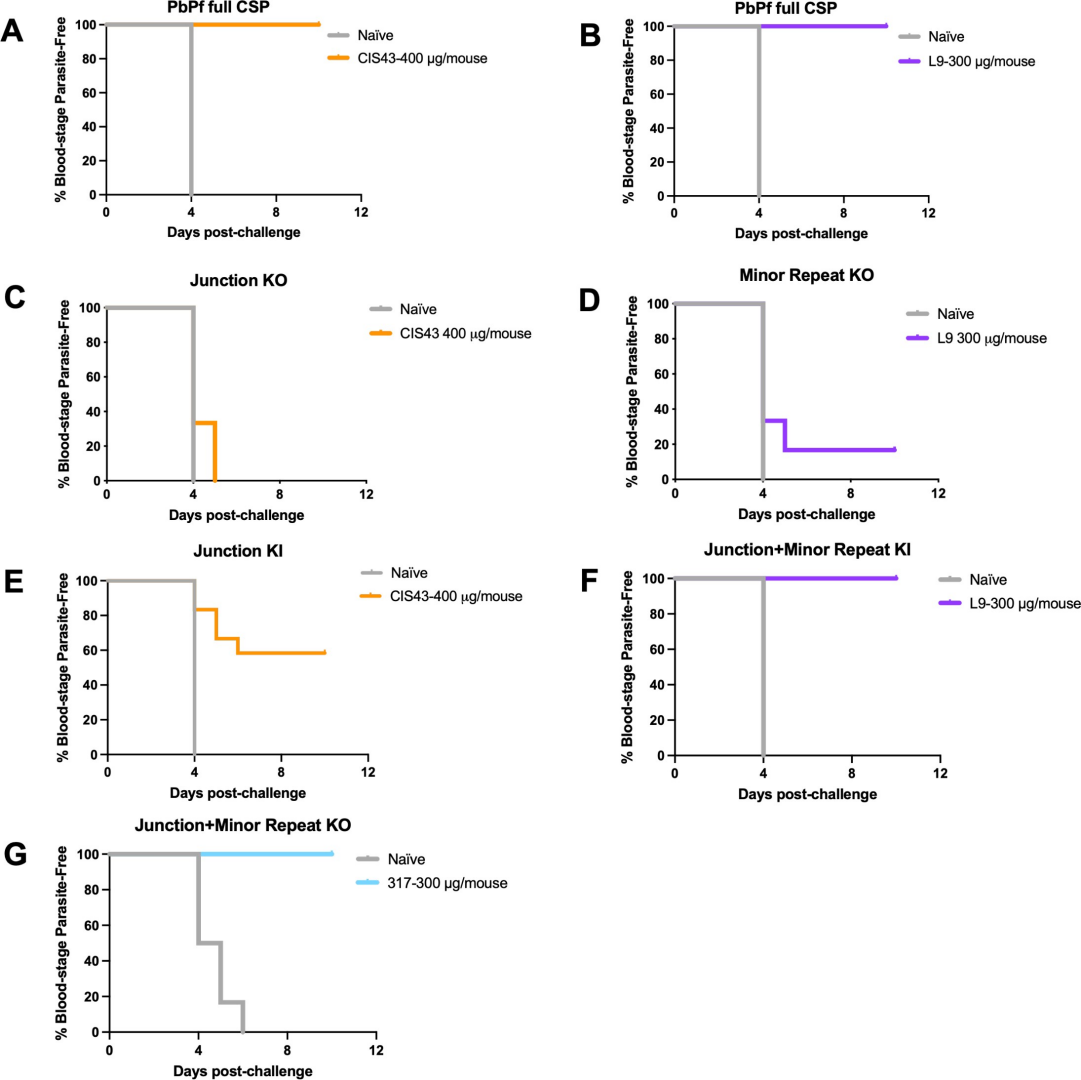
PfCSP mAb-mediated sterile protection in mice bitten by mosquitoes infected with transgenic SPZ lines. Kaplan-Meier survival curves of C57BL/6 mice (n = 12/group; combined from two independent experiments) injected with PBS (naïve), CIS43 (400 μg), L9 (300 μg), or 317 (300 μg) and challenged 16 hours later with 5 bites from mosquitoes infected with SPZ expressing (A-B) PbPf full CSP, (C) Junction KO, D) Minor Repeat KO, E) Junction KI, (F) Junction+ Minor Repeat KI, and G) Junction + Minor repeat KO.

To extend the *in vivo* protection analysis, we next evaluated the ability of CIS43 (400 μg), L9 (300 μg), and 317 (300 μg) to sterilely prevent SPZ from invading the liver and developing into blood-stage parasites in mice bitten by mosquitoes infected with the transgenic KO/KI SPZ lines. As a positive control, 100% of mice that received CIS43 or L9 16 hours before mosquito bite challenge with PbPf full CSP SPZ were sterilely protected (Fig 5A and 5B), consistent with prior studies [7,9].

Protection mediated by CIS43 (0%) and L9 (17%) was completely or substantially abrogated against the JR and MR KO SPZ, respectively (Fig 5C and 5D). Remarkably, protection mediated by CIS43 (60%) and L9 (100%) was largely restored against the JR KI and JR+MR KI SPZ, respectively (Fig 5E and 5F). Additionally, 317 provided 100% sterile protection against the JR+MR KO SPZ (Fig 5G), a high level of protection that was consistent with past PbPf full CSP SPZ mosquito bite challenge data [9]. These results are fully consistent with the i.v. challenge protection data (Figs 4, S2 and S3) and confirm that the binding of CIS43, L9, and 317 to their preferred JR, MR, and CR epitopes in the PfCSP repeat region is necessary and sufficient for these mAbs to confer protection against natural SPZ transmission from infected mosquitoes.

## Discussion

This study sought to determine whether highly potent human mAbs against the PfCSP repeat region mediate protection against SPZ challenge *in vivo* by preferentially binding their primary epitopes (junction region JR, minor repeats MR, or central major repeats CR) and/or by cross-reacting with secondary repeat epitopes. To assess this, transgenic Pb parasite lines expressing modified PfCSP lacking JR or MR (KO SPZ) and other lines expressing modified PbCSP containing short PfCSP JR and MR sequences (KI SPZ) were generated (Fig 1A, 1B, 1C, 1D, 1E, 1F and 1G). Previous studies have shown that modifications of CSP (e.g., deletion of the N-terminus, mutations in the N-terminal PEXEL/VTS motif, or complete deletion of CSP) disrupt or abrogate sporozoite infectivity [18–20].

However, all transgenic SPZ lines generated in this study retained an intact capacity to infect mice and mosquitoes similar to the PbPf full CSP control parasite line (Fig 1H and 1I and S3 Table), suggesting that mutating the junction or minor repeats of PfCSP does not impact the *in vivo* infectivity of transgenic Pb parasites.

Challenging mice with the transgenic KO/KI SPZ lines (Figs 4 and 5) clearly demonstrated that the three potent human mAbs used in this study require three distinct preferred epitopes in the repeat region of PfCSP to mediate *in vivo* protection. Specifically, protection mediated by anti-JR mAb CIS43 and anti-MR mAb L9 was strongly reduced when mice were challenged with KO SPZ expressing PfCSP that respectively lacked JR and/or MR but contained CR. As a corollary, CIS43- and L9-mediated *in vivo* protection was largely restored when mice were challenged with KI SPZ expressing short PfCSP JR and MR sequences inserted into PbCSP, which lacks CR. In contrast, *in vivo* protection mediated by the anti-CR mAb 317 was dependent on the presence of CR and was largely unaffected by the absence or presence of JR and MR. Interestingly, these data suggest that mAb cross-reactivity to secondary repeat epitopes has modest to no effect on SPZ neutralization. Overall, this study provides clear *in vivo* evidence that categorizes protective human PfCSP repeat mAbs into three distinct classes based on their preferential recognition of three antigenic determinants (junction region JR, minor repeats MR, and central major repeats CR) in the PfCSP repeat region.

There were several notable discrepancies between mAb binding to the transgenic KO/KI SPZ lines *in vitro* and protection *in vivo* (Figs 2 vs 4 and 5). For instance, CIS43 and 317 had high binding to JR KO SPZ (lack JR) and JR+MR KI SPZ (lack CR), respectively, but mediated minimal *in vivo* protection against each SPZ line. In contrast, the L9 binding and protection data against the transgenic SPZ lines was largely consistent. Specifically, L9 had lower *in vitro* binding and *in vivo* protection against JR+MR KO and JR KI SPZ (contain only one NPNV motif) but high binding and protection against JR+MR KI SPZ (contains two NPNV motifs), suggesting that L9 requires two adjacent NPNV motifs to potently bind and neutralize SPZ *in vivo*. Collectively, these data suggest that *in vitro* peptide, full-length protein, and SPZ binding assays—while useful for defining the specific PfCSP epitopes bound by mAbs [7,9,10]—may not necessarily predict their functional relevance *in vivo*.

After binding to CSP, protective antibodies can functionally neutralize SPZ *in vivo* by multiple mechanisms including immobilizing SPZ in the skin [17], killing SPZ in the skin and liver by stripping CSP from their surface, and preventing SPZ from invading hepatocytes [9,16]. Here, we measured the ability of the mAbs to immobilize the transgenic KO/KI SPZ lines *in vitro* and showed that this inhibitory phenotype largely correlated with *in vivo* protection. Importantly, these results show that CIS43 and L9 can immobilize SPZ by binding single epitopes.

An important aspect of using mAbs to passively prevent malaria is the degree to which their target epitopes are conserved. The JR (NPDPNANPNVDP), MR (NANPNVDPNANPNVDP), and CR (NANP)n epitopes are present in 99–100% of all *P*. *falciparum* field isolates sequenced to date. The major difference between isolates is the variation in the number of NANP and NVDP repeats (35–41 and 3–5, respectively) [21–23]. The high conservation of these three repeat epitopes suggests that using anti-PfCSP human mAbs for malaria prophylaxis may not be limited by parasite escape mutations in the field.

In terms of the clinical implications of these findings, CIS43 has completed Phase 1 testing [24]. The results of this study indicated that this anti-JR mAb can safely and effectively prevent Pf malaria following controlled exposure to infected mosquitoes and confirm that the JR is required for protection in humans. With regards to vaccine development, the current leading PfCSP-based malaria vaccine (RTS,S) contains only the PfCSP CR and C-terminus. For future research, our findings suggest that protection mediated by PfCSP-based vaccines can potentially be enhanced by including the JR and MR to increase the breadth of antibody responses. Finally, it will be important to determine if antibodies targeting these three distinct repeat epitopes (whether passively transferred as mAbs or induced by vaccination) have additive or antagonistic effects on *in vivo* protection.

## Material and methods

### Ethics statement

All assays involving mice were carried out in accordance with the recommendations in the Guide for the Care and Use of Laboratory Animals of the National Institutes of Health. Protocol number MO18H419, approved by the Animal Care and Use Committee of the Johns Hopkins University.

### Mice

6-7-week-old female C57BL/6 mice (Charles River Laboratories) were maintained at the animal facility of the Bloomberg School of Public Health, Johns Hopkins University. Every procedure performed was approved by the Animal Care and Use Committee (Protocol MO18H419).

### Monoclonal antibodies

Monoclonal antibodies were obtained as previously described [9]. Briefly, the sequences of CIS43 [7], L9 [9], 317 [6], VRC01 [25], and control antibody [12] were retrieved from PDB or GenBank and cloned into the pVRC8400 huIgG1, pVRC8400 huIgK, or SBShuLambda expression vectors (GenScript) containing the relevant constant region. Matched heavy and light chain constructs were co-transfected into Expi293 cells using the ExpiFectamine 293 Transfection Kit (Thermo Fisher Scientific) and cultures were incubated at 37°C, 8% CO_2_ for 6 days. Supernatants were harvested and purified using rProtein A Sepharose Fast Flow resin (GE Healthcare) and buffer exchanged with 1X PBS (pH 7.4) before being concentrated using Amicon Centrifugal Filters (Millipore) and sterile filtered through a 0.2 μm Steriflip filter units (Millipore). Purified mAbs were diluted to appropriate concentrations in sterile 1X PBS and concentrations were confirmed using a Nanodrop spectrophotometer.

### Generation of transgenic *P*. *berghei* parasite lines expressing PfCSP KO and KI sequences

Pb parasites (strain ANKA, 676m1c11, MRA-868) expressing green fluorescent protein (GFP) and luciferase were obtained from MR4, ATCC. Pyrimethamine plasmids (pR-CSPFL) encoding the different versions of PfCSP or PbCSP, along with the PbCSP signal sequence, were obtained from Bio Basic Inc. Plasmid transfections were performed as previously described [26] to generate the different transgenic parasites lines. All plasmids include the hDHFR selection cassette and *csp* 5’ and 3’ UTRs. pR-CSPFL was excised with XhoI and KasI and transfected into schizont cultures of Pb parasites by electroporation using an Amaxa Nucleofactor [13]. Transfected parasites were injected i.v. into Swiss Webster mice and selected with pyrimethamine dissolved in their drinking water (7 mg/mL). The pyrimethamine-resistant parasites were cloned by limiting dilution in mice, and isolated clones were verified by PCR and sequencing.

### Parasites

SPZ from Pb expressing GFP, luciferase, and the different CSP versions were obtained from salivary glands of infected mosquitoes, as previously described [12]. Briefly, *Anopheles stephensi* mosquitoes were fed on parasite-infected mice after confirming the presence of gametocyte exflagellation [27]. After infection, mosquitoes were maintained in an incubator at 19–20°C and supplied with a sterile cotton pad soaked in 10% sucrose, changed every 48 hrs. SPZ were harvested 20–22 days after blood feeding.

### Measurement of mAb binding to transgenic SPZ

Salivary glands containing SPZ were dissected as previously described [12]. The freshly harvested sporozoites were counted in a hemocytometer and placed on ice. Freshly harvested SPZ were stained with SYBR Green (10,000X concentrate; Thermo Fisher Scientific) diluted 1:2,000 in phosphate-buffered saline (PBS) for 30 min at 4°C, washed twice, and ~8,000 SPZ were aliquoted to each well of a 96-well V-bottom plate (50 μl/well). SPZ were incubated for 30 min at 4°C with anti-PfCSP or isotype control mAbs in PBS + 10% fetal bovine serum (PBS-FBS), washed twice with 200 μl PBS-FBS, stained for 20 minutes at 4°C with goat anti-human IgG-Alexa Fluor 647 secondary antibody (Thermo Fisher Scientific) diluted 1:1,000 in PBS-FBS, washed once with 200 μl PBS-FBS, and fixed in 200 μL PBS + 0.5% paraformaldehyde. Following fixation, events were acquired on a modified LSR II (BD Biosciences) and analyzed using FlowJo v.10.

### mAb-mediated inhibition of SPZ motility *in vitro*

The *in vitro* motility assays were performed using freshly dissected SPZ suspended in Hanks balanced salt solution (HBSS) containing 2% FBS. The parasite suspension was adjusted to a concentration of 10,000 SPZ/μL and 20 μL were allowed to settle on a 35 mm glass bottom Mattek dish for 10 minutes on ice. Motility was subsequently assayed using time-lapse microscopy at 37°C, with recordings made using an Olympus IX-71 inverted wide-field fluorescence microscope with a PCO complementary metal-oxide-semiconductor (CMOS) camera driven by DeltaVision software (Applied Precision, Seattle, WA). SPZ were imaged using an LED light source and FITC filter to visualize GFP fluorescence stably expressed by all parasite lines. A field of view containing approximately 30 SPZ was chosen, and a video was taken using a 5 second time-lapse for 20 minutes total. mAbs, dialyzed and re-suspended in HBSS with 2% FBS, were added directly to the dish containing the SPZ suspension to a final concentration of 25 μg/mL, unless otherwise indicated. Parasites continuously moving in a circle before mAb addition were identified as motile, and as non-motile only if their circular movement came to a complete stop 15 minutes after mAb exposure. Motility was assessed with the assistance of ImageJ software. Percentage of non-motile parasites were calculated as follows: non-motile SPZ / motile SPZ x 100.

### Sporozoite i.v. challenge

Estimation of parasite liver burden by bioluminescence was performed as previously described [12]. Briefly, transgenic SPZ freshly harvested from mosquito salivary glands were suspended in 2% FCS-HBSS media and adjusted to 10,000 SPZ/ml. Unless otherwise noted, mice were challenged with 2,000 SPZ i.v. in the tail vein, 16 hours after passive immunization of mAbs by i.v. injection into the tail vein. 42 hours after SPZ challenge, mice were injected intraperitoneally with 100 μL of D-Luciferin (30 mg/mL) and anesthetized in an isoflurane chamber. Once mice were immobilized, bioluminescence in their livers was measured for 5 minutes using an IVIS Spectrum in vivo imaging system (PerkinElmer).

### Mosquito bite challenge

Mosquito bite challenge in mice was performed as previously described [12]. Briefly, 16 hours after passive transfer of mAbs into the tail vein i.v., mice were exposed to the bites of five infected mosquitoes obtained from a population of mosquitoes that was 80% infected. Mice were anesthetized with 2% Avertin and placed on top of cages containing the infected mosquitoes for 10 minutes. Starting four days after mosquito bite challenge, Giemsa-stained blood smears were examined daily by light microscopy to look for blood-stage parasites until twelve days post-challenge.

## Supporting information

S1 Fig PfCSP mAb-mediated liver burden reduction in mice challenged i.v. with wild-type PbSPZ. C57BL/6 mice (4/group) were injected i.v. with PBS or 300 μg of mAbs CIS43, L9, 317, 3D11 (protective anti-PbCSP major repeat mouse mAb), or isotype control and i.v. challenged 16 hours later with 2,000 wild-type PbSPZ expressing PbCSP. Upper panel: parasite bioluminescence in the liver (total flux, photons/sec); lines indicate geometric mean and geometric standard deviation. Lower panel: fold reduction in liver burden mediated by each mAb, calculated as the ratio of the total flux in the PBS control mice divided by total flux in each mAb group.
(TIF)

S2 Fig Statistical analysis of PfCSP mAb-mediated liver burden reduction in mice challenged i.v. with transgenic SPZ lines. Parasite bioluminescence in the liver (total flux, photons/sec; lines indicate geometric mean and geometric standard deviation) in C57BL/6 mice (5/group) injected i.v. with PBS or 100 μg of mAbs CIS43, L9, or 317 and i.v. challenged 16 hours later with 2,000 transgenic SPZ. For each plot, PbPf full CSP SPZ was compared to SPZ expressing A) Junction KO, B) Junction KI, C) Minor Repeat KO, D) Junction+ Minor Repeat KI, and E) Junction + Minor repeat KO using the two-tailed Mann-Whitney test for PBS or each mAb. ns, not significant, p>0.05; *p  = 0.03; **p  = 0.0079.
(TIF)

S3 Fig Dose titration of PfCSP mAb-mediated liver burden reduction in mice challenged i.v. with transgenic SPZ lines. C57BL/6 mice (5/group) were injected i.v. with PBS or 300, 100, or 30 μg of CIS43, L9, 317 and i.v. challenged 16 hours later with 2,000 transgenic SPZ expressing A) PbPf full CSP, B) Junction KO, C) Junction KI, D) Minor Repeat KO, E) Junction+ Minor Repeat KI, and F) Junction + Minor repeat KO. Upper panels: parasite bioluminescence in the liver (total flux, photons/sec); lines indicate geometric mean and geometric standard deviation. Lower panels: fold reduction in liver burden mediated by each mAb, calculated as the ratio of the total flux in the PBS control mice divided by total flux in each mAb group.
(TIF)

S1 Table Epitope specificity of three potent human PfCSP mAbs. Name, preferred primary epitope, secondary epitopes, source from which they were isolated, and references for the three mAbs used in this study.
(XLSX)

S2 Table Modified CSP sequences in transgenic Pb parasite lines. Parasite name and selected sequence from the CSP repeat region detailing the modifications made to CSP (either PfCSP or PbCSP) expressed on the different transgenic Pb parasite lines generated in this study.
(XLSX)

S3 Table Infectivity of Pb transgenic parasite lines in mosquitoes and mice. Parasite name, percentage of mosquitoes with infected midguts, range of the number of oocysts per midgut, percentage of mosquitoes with infected salivary glands (SG), mean number of sporozoites, and percentage of mice that developed blood-stage parasitemia after exposure to five infected mosquito bites.
(XLSX)

S4 Table 50 percent maximal binding of mAbs to Pb transgenic parasite lines. The effective concentration required for 50% maximal binding (EC_50_, μg/mL) for each of the mAbs was calculated by using sigmoidal four-parameter nonlinear regression in Prism with log-transformed mAb concentration values.
(XLSX)

S1 Data Raw data used to generate the figures. (XLSX)
