# Supplementary figures and images for: The P. falciparum CSP repeat region contains three distinct epitopes required for protection by antibodies in vivo
Source: PLoS Pathog. 2021 Nov 8;17(11):e1010042. doi: 10.1371/journal.ppat.1010042 (PMC8601602; doi:10.1371/journal.ppat.1010042)

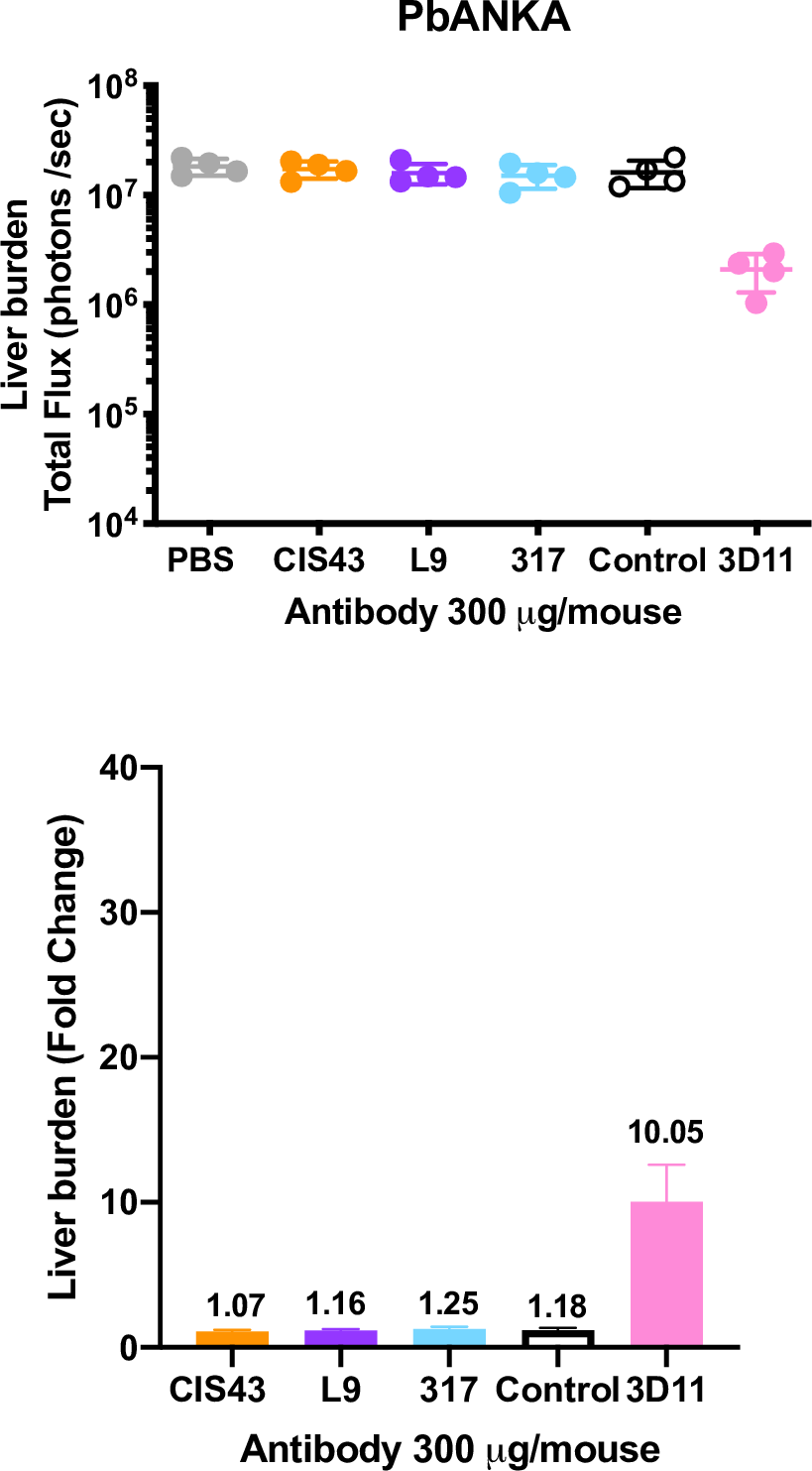

Supplement: S1 Fig — C57BL/6 mice (4/group) were injected i.v. with PBS or 300 μg of mAbs CIS43, L9, 317, 3D11 (protective anti-PbCSP major repeat mouse mAb), or isotype control and i.v. challenged 16 hours later with 2,000 wild-type PbSPZ expressing PbCSP. Upper panel: parasite bioluminescence in the liver (total flux, photons/sec); lines indicate geometric mean and geometric standard deviation. Lower panel: fold reduction in liver burden mediated by each mAb, calculated as the ratio of the total flux in the PBS control mice divided by total flux in each mAb group. (TIF) [file ppat.1010042.s001.tif]

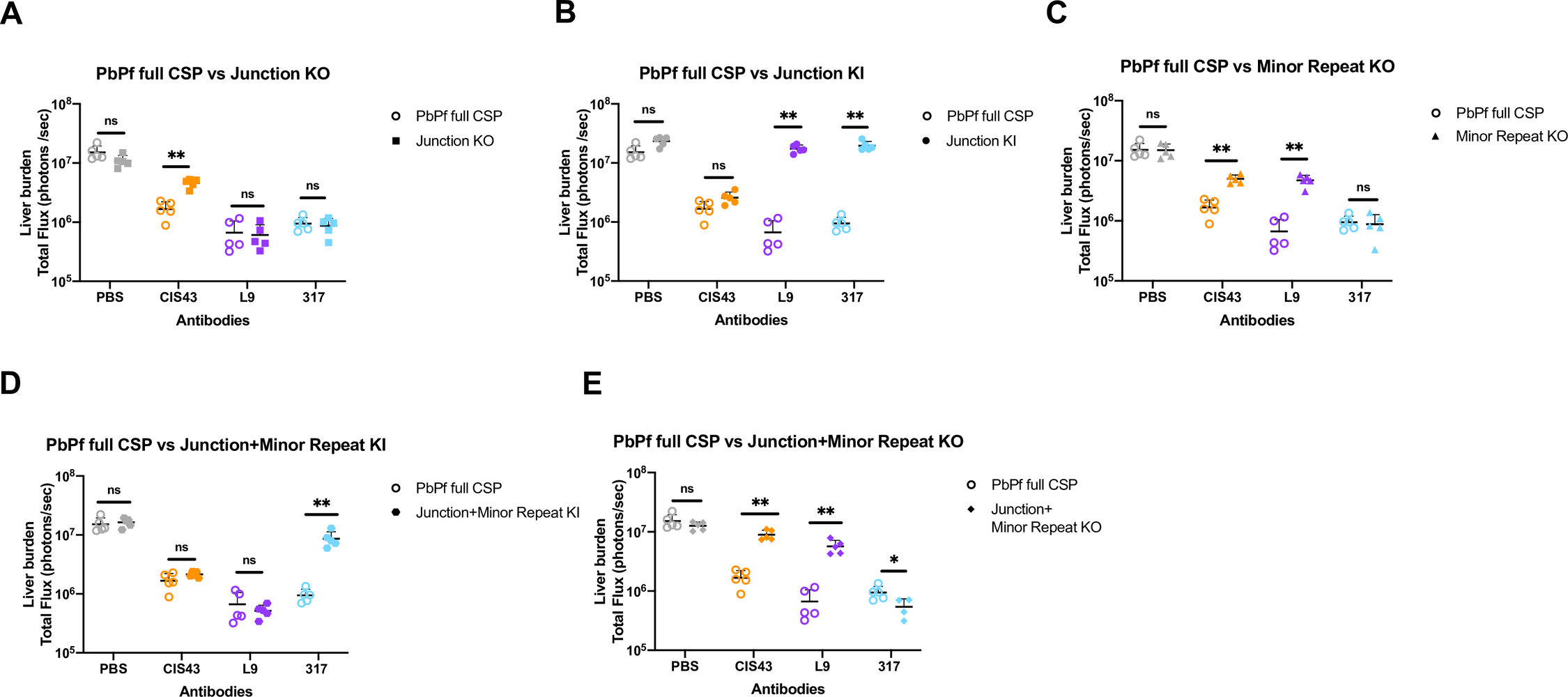

Supplement: S2 Fig — Parasite bioluminescence in the liver (total flux, photons/sec; lines indicate geometric mean and geometric standard deviation) in C57BL/6 mice (5/group) injected i.v. with PBS or 100 μg of mAbs CIS43, L9, or 317 and i.v. challenged 16 hours later with 2,000 transgenic SPZ. For each plot, PbPf full CSP SPZ was compared to SPZ expressing A) Junction KO, B) Junction KI, C) Minor Repeat KO, D) Junction+ Minor Repeat KI, and E) Junction + Minor repeat KO using the two-tailed Mann-Whitney test for PBS or each mAb. ns, not significant, p>0.05; *p = 0.03; **p = 0.0079. (TIF) [file ppat.1010042.s002.tif]

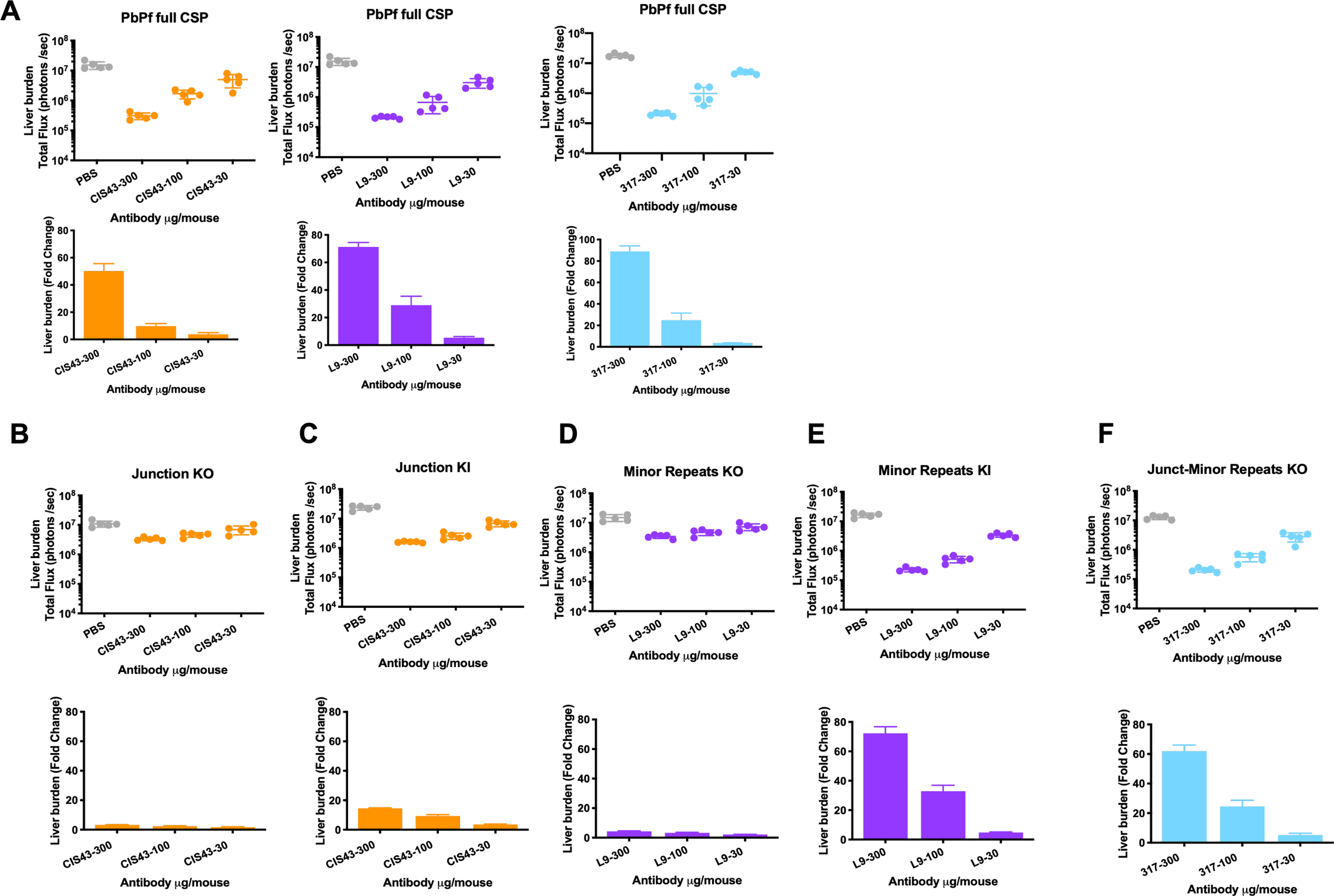

Supplement: S3 Fig — C57BL/6 mice (5/group) were injected i.v. with PBS or 300, 100, or 30 μg of CIS43, L9, 317 and i.v. challenged 16 hours later with 2,000 transgenic SPZ expressing A) PbPf full CSP, B) Junction KO, C) Junction KI, D) Minor Repeat KO, E) Junction+ Minor Repeat KI, and F) Junction + Minor repeat KO. Upper panels: parasite bioluminescence in the liver (total flux, photons/sec); lines indicate geometric mean and geometric standard deviation. Lower panels: fold reduction in liver burden mediated by each mAb, calculated as the ratio of the total flux in the PBS control mice divided by total flux in each mAb group. (TIF) [file ppat.1010042.s003.tif]
